# Close Relationship of Ruminant Pestiviruses and Classical Swine Fever Virus

**DOI:** 10.3201/eid2104.141441

**Published:** 2015-04

**Authors:** Alexander Postel, Stefanie Schmeiser, Tuba Cigdem Oguzoglu, Daniela Indenbirken, Malik Alawi, Nicole Fischer, Adam Grundhoff, Paul Becher

**Affiliations:** University of Veterinary Medicine, Hannover, Germany (A. Postel, S. Schmeiser, P. Becher);; Ankara Üniversitesi Veteriner Fakültesi Viroloji Anabilim Dalı Dışkapı, Ankara, Turkey (T.C. Oguzoglu);; Heinrich Pette Institute–Leibniz Institute for Experimental Virology, Hamburg, Germany (D. Indenbirken, M. Alawi, A. Grundhoff);; University Medical Center Hamburg-Eppendorf, Hamburg (M. Alawi, N. Fischer);; German Center for Infection Research Hamburg-Lübeck-Borstel, Hamburg (N. Fischer, A. Grundhoff);; German Center for Infection Research Hannover-Braunschweig, Hannover (P. Becher)

**Keywords:** novel pestivirus, CSFV, serology, CSF control, pestiviruses, classical swine fever virus, ruminant, viruses

## Abstract

To determine why serum from small ruminants infected with ruminant pestiviruses reacted positively to classical swine fever virus (CSFV)–specific diagnostic tests, we analyzed 2 pestiviruses from Turkey. They differed genetically and antigenically from known *Pestivirus* species and were closely related to CSFV. Cross-reactions would interfere with classical swine fever diagnosis in pigs**.**

Pestiviruses are enveloped viruses within the family *Flaviviridae* that have a highly variable single-stranded positive-sense RNA genome of ≈12.3 kb (*1*). The genus *Pestivirus* comprises the established species bovine viral diarrhea virus (BVDV)–1, BVDV-2, border disease virus (BDV), and classical swine fever virus (CSFV), as well as a growing number of additional tentative *Pestivirus* species. CSFV is the causative agent for classical swine fever, which is notifiable to the World Organisation of Animal Health because it is highly contagious and can cause great loss of pigs (*2*–*4*). For a given country, CSFV-positive status severely diminishes international trade of pigs and pig products. Accordingly, because of cross-reacting antibodies, infections of pigs (nonruminants) with ruminant pestiviruses, which occasionally occur under natural conditions, can cause serious problems with regard to serologic diagnosis of classical swine fever (*5*). 

In Turkey, 2 pestiviruses, Aydin/04 and Burdur/05, have been isolated from a sheep and a goat with clinical signs of border disease (*6*). A detailed genetic and antigenic characterization revealed that these 2 isolates must be regarded as representatives of a new *Pestivirus* species that is closely related to CSFV and can cause serious diagnostic problems in established CSFV serology.

## The Study

During 2004–2007, serum samples from 1,036 sheep and goats in Turkey were serologically screened for infection with pestiviruses of small ruminants. Of these, 11 serum samples from 7 sheep herds gave positive or doubtful reactions in the CSFV antibody–specific ELISA (HerdChek, IDEXX) and were subjected to commonly used virus neutralization testing (VNT) (*7*). VNT against the 2 established CSFV strains Alfort187 (genotype 1.1) and Diepholz (genotype 2.3) and against the BDV strains Moredun (genotype 1) and Gifhorn (genotype 3) revealed higher BDV titers in only 3 serum samples (Table 1). Equal or slightly higher titers against the CSFV reference strains became evident in 8 of the 11 serum samples, which came from 5 regions of Turkey. Further VNT analyses with the 2 previously obtained isolates, Aydin/04 and Burdur/05, demonstrated neutralizing antibody titers that were equal or higher than those against BDV and CSFV test strains. To elucidate the reason for strong serologic reactivity in CSFV assays, we genetically and antigenically characterized pestiviruses Aydin/04 and Burdur/05.

**Table 1:** Serologic pestivirus testing results for sheep from different districts in Turkey, 2004–2007*

Strain	CSFV†	Aydin	Burdur	BDV‡

Alfort187	Diepholz	Paderborn	Moredun	Frijters	X818	Reindeer	Gifhorn
Alfort187	1,810	452	113	**20**	**80**	5	10	≤3.5	10	12
Diepholz	381	1,280	452	**80**	**135**	57	28	10	48	28
Paderborn	34	160	452	**<3.5**	**14**	28	5	8.4	14	7,1
Aydin	**40**	**40**	**20**	**761**	**135**	**8.4**	**14**	**8.4**	**34**	**24**
Moredun	<3.5	4.2	<3.5	**<3.5**	**<3.5**	190	4.2	95	<3.5	<3.5
Frijters	14	20	17	**14**	**67**	113	761	269	134	12
X818	14	160	95	**24**	**30**	905	381	2,560	381	226
Reindeer	8.4	17	8.4	**4.2**	**17**	80	8.4	48	2,560	40
Gifhorn	14	80	67	**28**	**80**	30	40	67	134	5,120

*Boxes indicate virus neutralization test titers for homologous serum; boldface indicates results for Aydin/04 and Burdur/05. BDV, border disease virus; CSFV, classical swine fever virus. †CSFV strains: Alfort187 (genotype 1.1), Diepholz (genotype 2.3), Paderborn (genotype 2.1). ‡BDV strains: Moredun (genotype 1), Frijters (genotype 1), X818 (genotype 1), Reindeer (genotype 2), Gifhorn (genotype 3).

The complete genome sequence of Aydin/04 was determined as reported previously (*8*). The genome sequence of Burdur/05 was determined by next-generation sequencing on an Illumina MiSeq platform (2 × 250-bp paired end run, 593,328 reads) as recently described (*9*). Template total cellular RNA was extracted from supernatant of sheep fetal thymus cells. Of all reads, 73.9% were found to be of host origin. Of the nonhost reads, 89.9% assembled into a single sequence contig encompassing the entire pestivirus Burdur/05 genome (coverage 196-fold).

**Figure 1:**
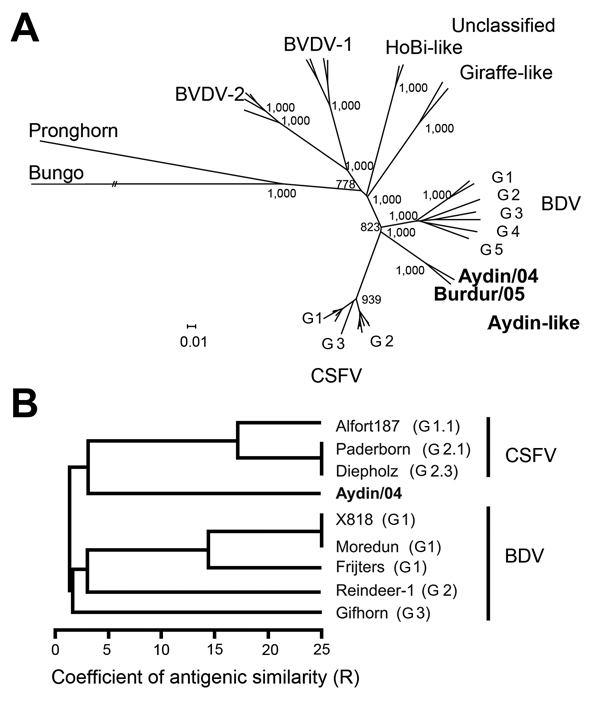
Phylogenetic and antigenic tree displaying relatedness of pestiviruses Aydin/04 and Burdur/05 to other *Pestivirus* species. A) For phylogenetic analysis, deduced polyprotein sequences from GenBank were used (CSFV: J04358, GU233734, JX218094, AY568569, GQ902941, KJ619377, AY382481, AF326963, X87939, AF099102, AY578687, AY646427; BDV: AF037405, U70263, KC963426, AF144618, GQ902940, KF918753, GU270877; BVDV-1: EF101530, AF220247, M96751, AF091605; BVDV-2: AB567658, GQ888686, AF502399, U18059; HoBi-like: AB871953, NC012812; giraffe-like: NC003678, KJ660072; Pronghorn and Bungowannah: NC024018, NC023176). Bootstrap values were calculated for 1,000 iterations. Only significant bootstrap values (≥700) of major nodes are given in the tree. Trees were displayed by using Dendroscope (*10*). Scale bar indicates base substitutions per site. B) Antigenic tree based on coefficients of antigenic similarity (R values) displaying antigenic relatedness of pestiviruses Aydin/04 and Burdur/05 to representative CSFV and BDV strains. R values <25 indicate significant antigenic differences as representing >4-fold differences in titers. R values >25 are considered not significant and are therefore not drawn to scale. Boldface indicates pestiviruses circulating among sheep and goat herds in Turkey. BDV, border disease virus; BVDV, bovine viral diarrhea virus; CSFV, classical swine fever virus; G, genotype.

Sequence and phylogenetic analyses were performed with complete genome sequences and deduced amino acid sequences of new pestiviruses Aydin/04 (GenBank accession no. JX428945) and Burdur/05 (KM408491). For further analyses, reference sequences were obtained from GenBank (Figure 1). Genetic distances were calculated by using the Kimura 2-parameter substitution model, and phylogenetic analyses were conducted by applying the neighbor-joining method as commonly used for CSFV phylogeny (*11*). With the same set of sequences, a grouping scan was performed by using the SSE platform (*12*). Comparison of the complete coding sequences of Aydin/04 and Burdur/05 revealed a genetic distance of 16.5%. Phylogenetic analyses based on deduced polyprotein sequences showed that isolates Aydin/04 and Burdur/05 form a distinct group located between CSFV and BDV (Figure 1, panel A).

Systematic antigenic characterization was performed by using cross-neutralization assays (Table 2). For this purpose, CSFV and BDV reference strains for which homologous serum was available were tested by VNT as described (*7*). In general, neutralization of both isolates was more efficient when performed with different CSFV antiserum than with BDV antiserum. In addition, the Aydin-specific antiserum obtained from animal experiments neutralized the CSFV reference strains with titers higher than those for the BDV strains (Table 2). Because no experimental infection with Burdur/05 has been performed, Burdur/05-specific antiserum was not available; however, close antigenic relatedness of both isolates was demonstrated by the high neutralization titers of the Aydin-specific antiserum for isolate Burdur/05 (Table 2). To quantify and to depict the antigenic relatedness, we calculated coefficients of antigenic similarity (R values) as described previously (*13*). An antigenic tree graphically displaying the R values clearly shows 2 distinct clades, one representing CSFV and the other comprising BDV strains (Figure 1, panel B). Furthermore, Aydin/04 is antigenically more closely related to CSFV than to BDV, but it also clearly differs from these 2 pestivirus species.

**Table 2:** Antigenic relationships determined by cross-neutralization of serum raised against different CSFV and BDV reference strains*

Strain	CSFV†	Aydin	Burdur	BDV‡

Alfort187	Diepholz	Paderborn	Moredun	Frijters	X818	Reindeer	Gifhorn
Alfort187	1,810	452	113	**20**	**80**	5	10	≤3.5	10	12
Diepholz	381	1,280	452	**80**	**135**	57	28	10	48	28
Paderborn	34	160	452	**<3.5**	**14**	28	5	8.4	14	7,1
Aydin	**40**	**40**	**20**	**761**	**135**	**8.4**	**14**	**8.4**	**34**	**24**
Moredun	<3.5	4.2	<3.5	**<3.5**	**<3.5**	190	4.2	95	<3.5	<3.5
Frijters	14	20	17	**14**	**67**	113	761	269	134	12
X818	14	160	95	**24**	**30**	905	381	2,560	381	226
Reindeer	8.4	17	8.4	**4.2**	**17**	80	8.4	48	2,560	40
Gifhorn	14	80	67	**28**	**80**	30	40	67	134	5,120

*BDV, border disease virus; CSFV, classical swine fever virus; boxes indicate virus neutralization test titers for homologous serum; boldface indicates results for Aydin/04 and Burdur/05. †CSFV strains: Alfort187 (genotype 1.1), Diepholz (genotype 2.3), Paderborn (genotype 2.1). ‡BDV strains: Moredun (genotype 1), Frijters (genotype 1), X818 (genotype 1), Reindeer (genotype 2), Gifhorn (genotype 3).

Because of their close relationship to CSFV, it was of particular interest to determine the ability of these ruminant pestiviruses to infect pigs and induce clinical disease. Therefore, 3 clinically healthy and pestivirus uninfected weaner (6 weeks of age) piglets were inoculated with 1 × 10^6^ 50% tissue culture infectious doses of isolate Aydin/04 and given a booster of 3 × 10^7^ 50% tissue culture infectious doses 2 weeks later. Pigs showed no clinical signs of disease, no fever, no platelet or leukocyte depletion, and no viremia (data not shown). For all 3 animals, strong seroconversion was found (50% neutralizing titer of serum for homologus virus was 240–640 on postinoculation day 77).

## Conclusions

**Figure 2:**
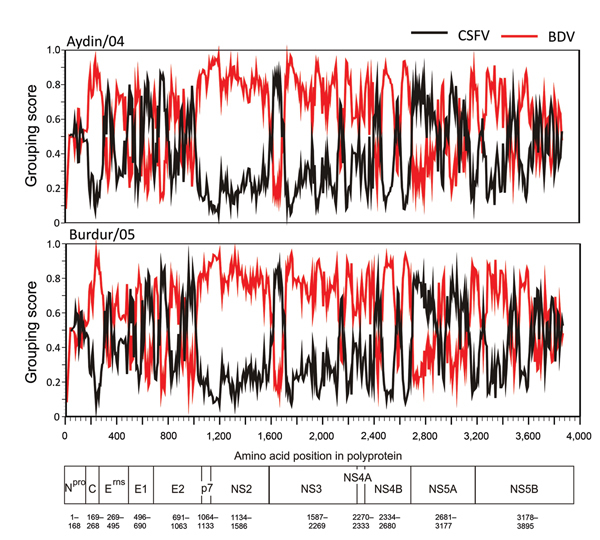
Amino acid similarity of pestiviruses Aydin/04 and Burdur/05 to representative CSFV and BDV polyprotein sequences. The same CSFV and BDV polyprotein sequences as in Figure 1 were used for analysis. Grouping scan was performed with the SSE software platform as described previously, by using a window of 200 aa with 20-aa increments (*12*). For calculation of genetic distances, the Kimura 2-parameter model was applied. Borders of the mature viral proteins in the polyprotein of Aydin/04 are given below. BDV, border disease virus; CSFV, classical swine fever virus; C, core protein; E, envelope protein; rns, ribonuclease secreted; N^pro^, N-terminal autoprotease; NS, nonstructural protein; p7, protein p7.

Several new genetically diverse groups of pestiviruses have emerged in domestic livestock and wild animals, adding to the continuously growing list of approved and tentative pestivirus species (*1*). According to phylogenetic analyses of short partial genome sequences, 2 pestivirus isolates, Aydin/04 and Burdur/05, recently circulating in sheep and goat herds in different regions of Turkey, were classified as novel members of the BDV species (*6*). However, the data from this study demonstrate that these novel Aydin-like pestiviruses are representatives of a new pestivirus species, genetically and antigenically located between CSFV and BDV (Figure 1). The genetic distance of 16.5% between these isolates indicates that distinct ruminant Aydin-like pestiviruses circulate in different regions of Turkey. For some genomic regions, both ruminant pestivirus isolates display an even higher similarity to CSFV than to BDV (Figure 2). The close genetic relatedness to CSFV is in line with the antigenic characterization by cross-neutralization assays as depicted in the antigenic tree (Figure 1, panel B). This finding is in contrast with findings for pestivirus isolates from Tunisia, another group of ruminant pestiviruses genetically closely related to CSFV but antigenically more closely related to BDV (*14*). The close antigenic relationship to CSFV explains the observed strong cross-reactivity of serum from sheep and goat in CSFV-specific ELISAs, even when the variable E2 protein is used as diagnostic antigen (Table 1). In routine diagnosis, questionable ELISA results are further investigated by VNT against CSFV and other pestiviruses (e.g., BVDV and BDV). Usually, VNT titers are highest for the homologous pestivirus species. Remarkably, even if representatives of the Aydin-like pestiviruses were included as test strains in the VNT, CSFV infection still could not be ruled out.

Although these novel pestiviruses are the closest known relatives of CSFV, experimental infection of pigs with Aydin/04 did not result in detectable viremia and clinical signs. Nevertheless, these ruminant pestiviruses are candidates for a switch to porcine hosts after ongoing virus evolution, which would have severe consequences for serologic diagnosis of classical swine fever, affecting control and monitoring programs performed in many parts of the world.
